# Transvaginal Ultrasound for the Diagnosis of Endometriosis: Current Practices and Barriers in Australian Sonographers

**DOI:** 10.1002/ajum.70003

**Published:** 2025-05-22

**Authors:** Xinyu Yang, Alison Deslandes, Teresa Cross, Jessie Childs

**Affiliations:** ^1^ Allied Health and Human Performance Unit University of South Australia Adelaide Australia; ^2^ Robinson Research Institute University of Adelaide Adelaide Australia

**Keywords:** barriers, endometriosis, facilitators, gynaecology, sonographer, transvaginal ultrasound

## Abstract

**Introduction/Purpose:**

The 2016 consensus statement from the International Deep Endometriosis Analysis group (IDEA) outlined a transvaginal ultrasound (TVUS) approach specific for the sonographic assessment of endometriosis (eTVUS). However, eTVUS remains a nonroutine sonographic examination, and the reasons for this are not fully understood. This study aimed to explore the current performance of eTVUS among Australian sonographers and the barriers and facilitators encountered when learning and implementing eTVUS into routine practice.

**Methods:**

An online cross‐sectional survey was disseminated to Australian sonographers. Quantitative and qualitative questions were asked regarding demographic information, eTVUS performance and experiences encountered when learning and implementing eTVUS. Statistical and thematic analyses were performed.

**Results:**

In total, 127 responses were analysed, with 47.8% of respondents performing a full or partial eTVUS routinely. When a gynaecological ultrasound is referred, 18.4% of participants reported performing a full assessment of eTVUS, and 29.8% reported performing a partial assessment of eTVUS. When a partial eTVUS was performed, respondents indicated this mostly included an assessment of the sliding sign (94.6%) and ovarian mobility (97.3%), rather than a search for endometriotic nodules. Only 41.5% of all participants reported confidence in performing eTVUS.

The main barriers that limited the uptake of eTVUS were limited supervision/mentors (42.3%), limited reporting of eTVUS (39.6%) and its steep learning curve (38.7%). The main facilitators included sonographers' desire to answer the clinical question for suspected endometriosis (84.0%), external education (38.7%), local department protocols (30.7%) and colleagues who perform eTVUS (30.7%).

**Conclusion:**

While eTVUS, or aspects of it, are being performed in most imaging practices, inconsistency exists for the anatomical structures assessed as part of an eTVUS. Although barriers exist, more education surrounding eTVUS for sonographers, reporting doctors, and referrers could help increase uptake into routine practice.

## 1 Introduction

Endometriosis is a common condition characterised by the presence of endometrium‐like epithelium and/or stroma outside the uterus [1]. It can be categorised into three main subtypes: superficial endometriosis (SE), ovarian endometriosis (OE) and deep endometriosis (DE) [1]. In Australia, it is estimated that one in seven women and individuals presumed female at birth are diagnosed with or suspected to have endometriosis by the age of 49 [2]. This chronic condition can severely affect an individual's quality of life, manifesting with symptoms such as dysmenorrhea, infertility and heavy menstrual bleeding [3]. In Australia, the diagnosis of endometriosis is delayed on average by 6.4 years from the onset of symptoms due to poor awareness and diagnostic challenges [4, 5].

Historically, laparoscopy was considered the gold standard for diagnosing endometriosis; however, it is invasive, carries surgical risk and accessibility can be limited [6]. Recent updated guidelines from the European Society of Human Reproduction and Embryology (ESHRE) [6] suggest that imaging techniques, particularly transvaginal ultrasound (TVUS), should replace laparoscopy as the first‐line investigation tool for suspected endometriosis due to its superior feasibility, ease of access and diagnostic accuracy. The ESHRE guidelines suggest that laparoscopy should only be used for diagnosis when imaging results are inconclusive, as ultrasound is unable to exclude all subtypes of endometriosis (e.g., SE and extra‐abdominal endometriosis) [1, 6].

A typical routine TVUS (rTVUS) as per the Australasian Society of Ultrasound in Medicine (ASUM) guidelines [7] for the performance of gynaecological ultrasound includes assessments of the uterus, cervix, ovaries, adnexa and basic status of the pouch of Douglas (POD). In 2016, a consensus statement proposed by the International Deep Endometriosis Analysis group (IDEA) outlined comprehensively what constitutes a comprehensive TVUS for deep endometriosis (eTVUS) [8]. Specifically, additional assessments of the pelvic anatomy were recommended to improve the detection rate of OE and DE during eTVUS examination [8, 9], detailed in Table 1.

**TABLE 1:** Anatomy assessed using routine transvaginal ultrasound (rTVUS) [7] versus transvaginal ultrasound specific to endometriosis diagnosis (eTVUS) [8, 9].

rTVUS [7]	eTVUS [8, 9]
– Uterus – Cervix – Ovaries – Adnexa – Pouch of Douglas ○ Fluid	– Uterus – Cervix – Ovaries – Adnexa – Pouch of Douglas ○ Fluid ○ Obliteration ○ Endometriosis nodules – Anterior compartment ○ Urinary bladder ○ Uterovesical region ○ Ureters – Posterior compartment ○ Posterior vaginal wall ○ Uterosacral ligaments ○ Rectovaginal septum ○ Rectosigmoid colon – Lateral compartment ○ Parametrium – Uterine sliding sign – Assessments of soft markers ○ Ovarian mobility ○ Site‐specific tenderness

Multiple studies have been conducted demonstrating the high diagnostic accuracy of eTVUS in detecting OE and DE in the anterior and posterior compartments [10, 11, 12]. A 2016 Cochrane review evaluated 17 studies and reported that eTVUS had a sensitivity of 93% and specificity of 96% for OE detection, and a sensitivity of 79% and specificity of 94% for DE detection [13]. A more recent systematic review by Deslandes et al. [14] suggested similar diagnostic performance of eTVUS, with overall higher sensitivity and lower specificity for DE detection.

Considering the prevalence of endometriosis and its associated diagnostic delay, coupled with the high diagnostic value of eTVUS, advocates have suggested the implementation of this technique into routine ultrasound practice [15, 16]. It is unknown exactly how many practices in Australia offer eTVUS or how many sonographers can perform the protocol satisfactorily. Anecdotally, however, despite the commonality of endometriosis, it has been noted that eTVUS is not routinely offered in many imaging centres within Australia.

Little evidence exists within the literature to explain why the uptake of eTVUS into routine practice may be slow. Studies have suggested that the potential barriers to the adoption of eTVUS within sonographic practice could include a steep learning curve [17], poor awareness of the technique among referring clinicians [18], and inadequate scanning time allocated by ultrasound departments [19]. Furthermore, studies have suggested that a dedicated training program and a more comprehensive, up‐to‐date protocol could facilitate the uptake and learning process of eTVUS [20, 21]. To date, no studies have assessed the firsthand experiences of Australian sonographers regarding the adoption of the eTVUS technique. The aim of this study was to investigate the capability of Australian sonographers in the use of eTVUS along with current sonographic practice for endometriosis diagnosis in Australia, as well as the barriers and facilitators they encountered when learning and implementing eTVUS in routine practice.

## 2 Methodology

### 2.1 Ethics Statement

Ethics approval was obtained for this study from the University of South Australia (UniSA) Human Research Ethics Committee (project number 205748).

### 2.2 Study Design

An anonymous online survey targeting Australian sonographers was conducted using REDCap electronic data capture tools hosted at UniSA [22, 23]. The survey comprised 40 questions that were devised based on literature and the guidance of subject matter experts (AD, TC, JC). Specifically, quantitative questions were developed to identify participant demographic information, the current sonographic practice for endometriosis diagnosis, and participants' capability in performing eTVUS. A mix of 12 quantitative and qualitative questions were used to capture participant experiences when learning and performing eTVUS, including four questions focussed on sonographers' perceived barriers and facilitators when implementing eTVUS into routine practice. A full list of survey questions can be seen in Appendix S1.

Following the construction of the survey, three experienced sonographers were invited to assess the survey questions to ensure face and content validity. Minor modifications to wording and formatting were implemented based on the feedback received. A participant information sheet was provided on the front page of the online survey, and participants were required to tick the ‘I consent’ box to provide informed consent and commence the survey. The survey was open from 23rd October 2023 to 31st January 2024 inclusive.

A convenience sample was recruited by distributing the survey through social media groups aimed at sonographers, posters placed in ultrasound departments, as well as through the eNewsletters of the Australasian Society for Ultrasound in Medicine (ASUM) and the Australian Society of Medical Imaging and Radiation Therapy (ASMIRT). Accredited medical or student sonographers over the age of 18 years who perform gynaecological ultrasounds in Australia were eligible to participate. Individuals involved in the development and validity testing of the survey were excluded. To achieve a 90% confidence interval with a 10% margin of error, based on the total population size of 8370 Australian sonographers, a minimum sample size of 67 responses was deemed necessary [24, 25].

### 2.3 Statistical Analysis

Quantitative results were analysed using descriptive statistics. Fisher's exact test was employed to examine any associations between the data and demographics [26]. A *p* value of ≤ 0.05 was considered statistically significant [27]. Counts and percentages were used for categorical variables.

The open‐ended questions yielded qualitative, free‐text nominal data, which was analysed using a reflexive thematic analysis approach conducted by the principal investigator [28]. Each analysis was repeated by at least one member of the research team. Any disagreements were resolved through verbal discussion until consensus was reached.

## 3 Results

### 3.1 Participant Demographics

A total of 151 responses to the survey were received. After excluding 24 blank responses, 101 complete and 26 partially complete responses were included in the final analysis. Demographic information for participants is outlined in Table 2, and participant experiences and workplace environment are outlined in Table 3.

**TABLE 2:** Participant demographics.

Variable	Response	*n*, (%)
Age *n* = 127	18–24 years	6, (4.7%)
	25–34 years	37, (29.1%)
	35–44 years	37, (29.1%)
	45–54 years	29, (22.8%)
	55–64 years	14, (11.0%)
	Over 65 years	4, (3.1%)
	Prefer not to say	0, (0.0%)
Gender *n* = 127	Male	20, (15.7%)
	Female	107, (84.3%)
	Transgender	0, (0.0%)
	Nonbinary	0, (0.0%)
	Prefer not to say	0, (0.0%)
State/territory of practice *n* = 127	Australian Capital Territory	3, (2.4%)
	New South Wales	31, (24.4%)
	Northern Territory	2, (1.6%)
	Queensland	29, (22.8%)
	South Australia	15, (11.8%)
	Tasmania	5, (3.9%)
	Victoria	31, (24.4%)
	Western Australia	11, (8.7%)
Employment sector *n* = 125	Public hospital: general radiology department	30, (24.0%)
	Public hospital: Obstetrics and Gynaecology imaging department (or equivalent)	5, (4.0%)
	Private clinics: general radiology department	18, (14.4%)
	Private hospital: Obstetrics and Gynaecology imaging department (or equivalent)	6, (4.8%)
	Private practice: general radiology	49, (39.2%)
	Private practice: Specialist obstetrics and gynaecological imaging	15, (12.0%)
	Other—Public hospital, private practice	1, (0.8%)
	Other—Private hospital	1, (0.8%)
Employment status *n* = 125	Full‐time	59, (47.2%)
	Part‐time	53, (42.4%)
	Casual	12, (9.6%)
	Locum	1, (0.8%)

**TABLE 3:** Participant experience and workplace environment.

Variable	Response	*n*, (%)
Postgraduate experience *n* = 127	Student sonographer	16, (12.6%)
	Less than 2 years	7, (5.5%)
	2–5 years	19, (15.0%)
	6–10 years	17, (13.4%)
	11–15 years	18, (14.2%)
	Over 15 years	50, (39.4%)
Billing type of practice *n* = 125	Mostly bulk‐billing private practice	30, (24.0%)
	Mostly fee‐charging private practice	63, (50.4%)
	Public hospital	31, (24.8%)
	Other—Some bulk bill, some fee paying	1, (0.8%)
Number of TVUS performed per week *n* = 124	Less than 5 scans	12, (9.7%)
	6–10 scans	46, (37.1%)
	11–20 scans	43, (34.7%)
	More than 20 scans	23, (18.5%)
Report writers of TVUS scans performed by participants *n* = 124	General radiologist	88, (71.0%)
	Radiologist sub‐specialised in OBGYN imaging	13, (10.5%)
	OBGYN sonologist	16, (12.9%)
	Unsure	2, (1.6%)
	Other—Sonographer who performs the scan	2, (1.6%)
	Other—Radiologist and OBGYN sonologists	2, (1.6%)
	Other—Nuclear medicine physician with ultrasound training	1, (0.8%)

Abbreviations: OBGYN, Obstetrics and Gynaecology; TVUS, transvaginal ultrasound.

### 3.2 Current Sonographic Practice for Endometriosis Diagnosis; Capability of eTVUS Performance

**FIGURE 1:**
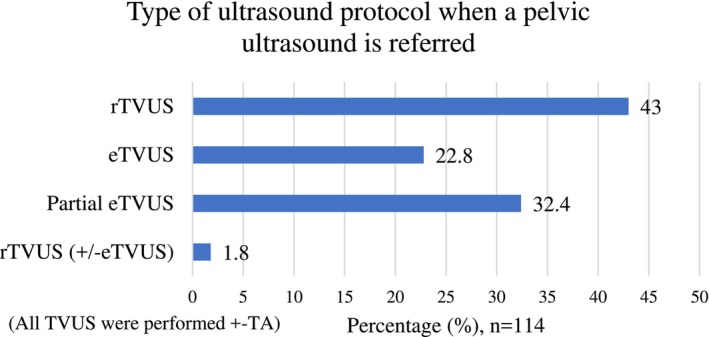
Type of ultrasound protocol when a pelvic ultrasound is referred (*n* = 114). eTVUS, transvaginal ultrasound specific for endometriosis detection; rTVUS, routine transvaginal ultrasound; TA, transabdominal ultrasound. Partial eTVUS, only some parts of the additional anatomical areas included in the eTVUS protocol are scanned.

When an ultrasound related to the pelvic region is referred, 43% (*n* = 49/114) of participants reported they would perform rTVUS with or without transabdominal ultrasound (TA) based on the department protocol (Figure 1). Regarding the performance of eTVUS, 22.8% (*n* = 24/114) of participants reported performing a full assessment of eTVUS (with or without TA), and 32.4% (*n* = 37/114) reported performing a partial assessment of eTVUS (with or without TA) (Figure 1).

**FIGURE 2:**
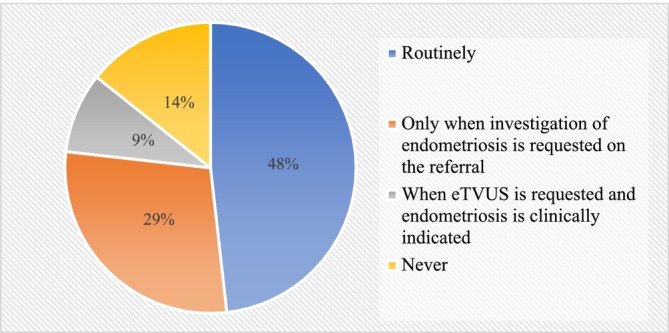
Frequency of performing a full or partial eTVUS when a gynaecological ultrasound is indicated (*n* = 113). ETVUS, transvaginal ultrasound specific for endometriosis diagnosis.

Specifically, when a TVUS is indicated, a complete or partial eTVUS protocol is routinely performed by approximately half of all the participants (47.8%, *n* = 54/113) (Figure 2). Additionally, 38.1% (*n* = 43/113) of participants reported performing eTVUS only under specific circumstances, such as when endometriosis investigation is requested in the referral, suspected based on the rTVUS scan or indicated by the patient's clinical history (Figure 2).

**FIGURE 3:**
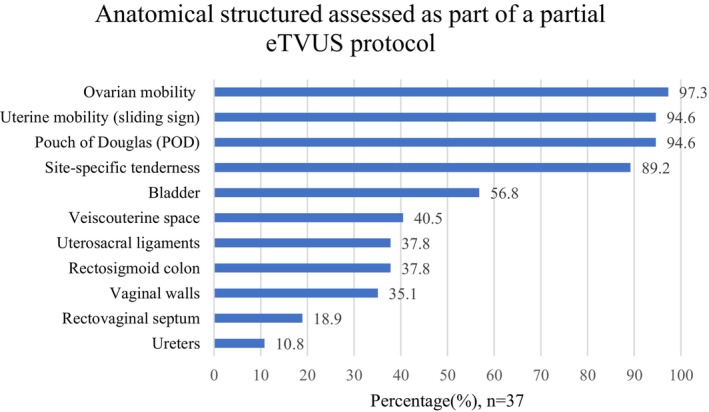
Anatomical structures assessed as part of a partial eTVUS protocol (participants could select more than one option) (*n* = 37). ETVUS, transvaginal ultrasound specific for endometriosis diagnosis; POD, pouch of Douglas.

When participants reported performing only a partial eTVUS protocol, the anatomy assessed varied between respondents (Figure 3). Ovarian mobility and the uterine sliding sign were assessed by 97.3% and 94.6% of those performing a partial eTVUS protocol, respectively. However, only 37.8% reported assessing the uterosacral ligaments (USLs) and rectosigmoid colon, and just 10.8% reported assessing the ureters (Figure 3).

Regarding the capability of performing eTVUS, less than half of the participants (41.5%, *n* = 44/106) reported being confident in performing the procedure. In addition, 9.9% (*n* = 11/111) of participants reported not being aware of eTVUS before participating in the survey, 24.3% (*n* = 27/111) were aware of eTVUS but did not know how to perform the complete assessment, and 22.5% (*n* = 25/111) were currently learning to perform eTVUS.

Participants working in specialised Obstetrics and Gynaecology (OBGYN) settings (81.0%, *n* = 17/21) were significantly more likely to be able to perform eTVUS than those working in general radiology settings (34.4%, *n* = 31/90) (*p* < 0.05). Moreover, participants who work in fee‐charging private practices (57.6%, *n* = 34/59) were significantly more likely to report being capable of performing eTVUS than participants who work in bulk‐billing private practices (23.8%, *n* = 5/21) or in public hospitals (19.2%, *n* = 5/26) (*p* < 0.05).

### 3.3 Theoretical Learning and Gaining Hands‐on Practice

Various strategies were reported by participants regarding the theoretical learning of eTVUS. The most common methods were attending conference presentations/workshops (61.7%, *n* = 65/107), online education through professional organisations (49.5%, *n* = 53/107), reading formal publications such as journal articles (48.6%, *n* = 52/107), learning from colleagues or workplace mentors (36.4%, *n* = 39/107) and free online education including YouTube videos (29.0%, *n* = 31/107).

A free‐text question was employed to capture information about participants' methods to gain hands‐on practice experience for the consolidation of their eTVUS skill. Self‐directed practice of eTVUS in routine scans was identified as a recurring theme across the responses:

(I would) do it on all patients regardless of the reason for the pelvic ultrasound to gain confidence. (Participant 95)

Another theme identified was participants were able to practice and consolidate their eTVUS skill under supervision of colleagues, mentors and experienced clinicians, with responses such as:

Specific mentors instructing me what images are important to take for detecting endo(metriosis). (Participant 20)

(A) dedicated experienced and motivated gynaecologist who stands beside (me) while I scan. (Participant 140)

### 3.4 Barriers

**FIGURE 4:**
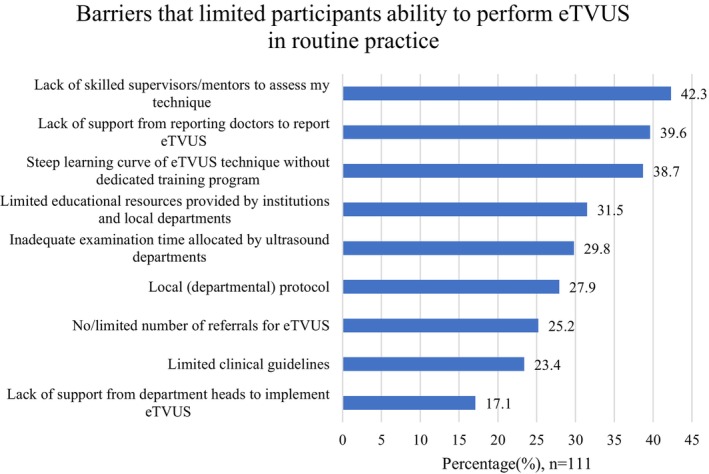
Barriers that limited participants ability to perform eTVUS in routine practice (participants could select more than one option) (*n* = 111). ETVUS, transvaginal ultrasound specific for endometriosis diagnosis.

The top three barriers that limited participants ability to perform eTVUS in routine practice were: lack of skilled supervisors/mentors to assess their technique (42.3%, *n* = 47/111), lack of support from reporting doctors to report eTVUS (39.6%, *n* = 44/111) and the steep learning curve of the eTVUS technique without a dedicated training program (38.7%, *n* = 43/111) (Figure 4). Notably, participants who work in general radiology departments (43.4%, *n* = 43/99) were more likely to report a lack of supervision to assess their technique as a barrier, compared with those working in OBGYN specialised departments (15.4%, *n* = 4/26) (*p* < 0.05). Furthermore, two male participants (1.8%, *n* = 2/111) reported concerns regarding consent and the medicolegal risk associated with performing eTVUS due to the dynamic nature of the examination.

### 3.5 Facilitators

**FIGURE 5:**
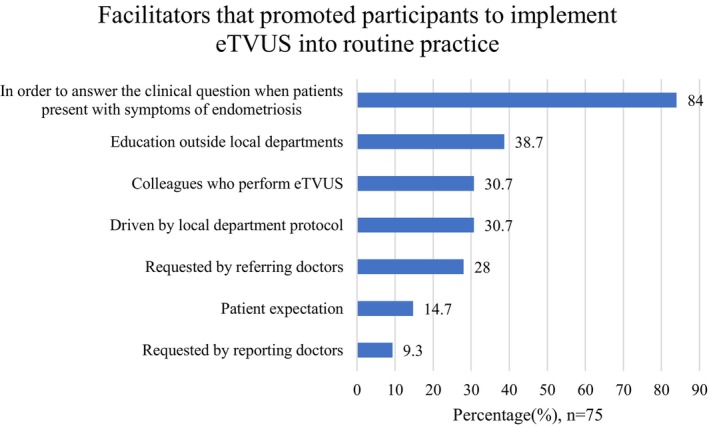
Facilitators that allowed participants to implement eTVUS into routine practice (participants could select more than one option) (*n* = 75). ETVUS, transvaginal ultrasound specific for endometriosis diagnosis.

The most reported facilitator that enabled participants to implement eTVUS into routine practice was a desire to answer the clinical question when patients present with symptoms of endometriosis (84.0%, *n* = 63/75) (Figure 5). Additionally, education outside local departments (38.7%, *n* = 29/75), local department protocols (30.7%, *n* = 23/75) and colleagues who perform eTVUS (30.7%, *n* = 23/75) were also identified as facilitators (Figure 5).

### 3.6 Departmental Support

Regarding support received from local departments, 39.8% (*n* = 41/103) of participants reported being encouraged to perform eTVUS in routine practice by their departments. Notably, participants who were encouraged by their departments to perform eTVUS (86.7%, *n* = 26/30) were significantly more likely to feel confident performing eTVUS than those who were not encouraged by their departments (39.0%, *n* = 16/41) (*p* < 0.05).

The methods of support that workplaces provided for sonographers to perform eTVUS were further investigated using a free‐text open‐ended question. Training of eTVUS provided by the department was identified as one of the main themes, with responses such as:

(Department provides) educational program … (Participant 99)

… mentoring to perform eTVUS. (Participant 58)

Another theme identified was the establishment of dedicated protocols and worksheet for eTVUS performance, with response:

Specific protocols within our department on when to perform eTVUS and when to perform rTVUS. (Participant 93)

Moreover, one recurring theme recognised by participants was the support they received from referring clinicians, reporting clinicians, and/or the chief sonographers of the department, with responses including:

(I am) Supported by reporting Drs & these Drs will come into the room to assist & educate on the cases we find difficult. (Participant 24)

Senior sono(grapher) encourages further training, has organized(sic) webinars and will check scans to ensure standards are met. (Participant 43)

DE scans are part of the bookings we receive, so we have to (be) competent in performing them. (Participant 89)

### 3.7 Reporting System

Respondents indicated that if they performed an eTVUS examination, the assessments (in addition to rTVUS) for endometriosis were always reported in 31.3% (*n* = 31/99), often reported in 20.2% (*n* = 20/99) and sometimes reported in 33.3% (*n* = 33/99). Most participants (90.0%, *n* = 18/20) working in OBGYN specialised departments indicated that the additional eTVUS assessments for endometriosis investigation they performed would always/often be reported, which was a significantly higher rate compared to participants working in general radiology departments (50.8%, *n* = 33/65) (*p* < 0.05). As an aid in reporting, 75.4% (*n* = 86/114) of practice protocols also instructed sonographers to save cine clips, in addition to still images, to fully document a gynaecological ultrasound.

A free‐text question was employed to collect insight from participants about the reporting system of the eTVUS images. Some participants suggested that increased confidence from the reporting clinicians in the sonographer's findings would be of benefit, with responses such as:

Sonographer comments on worksheet are mostly ignored. (Participant 26)

Drs are more likely to include mobility assessment in report when it appears normal. Very few Drs will make comment if restricted mobility is demonstrated, no matter how good the quality of scan is. (Participant 114)

Some other participants further suggested that more confident and specialised clinicians are needed to report eTVUS findings, with response noted:

We document mobility and suspicion of endo, some radiologists are quite good at adding detail, others keep comments to a minimum even when the appearances strongly suggest endometriosis. (Participant 147)

## 4 Discussion

This study was the first to investigate the capability of Australian sonographers in the use of eTVUS, along with current sonographic practice for endometriosis diagnosis in Australia, and the barriers and facilitators they encountered when learning and implementing eTVUS in routine practice. Approximately half of the participants (55.2%) reported performing a full or partial eTVUS when a gynaecological ultrasound is referred, while only 41.5% of all participants reported being confident performing eTVUS. Specifically, 22.8% of all participants reported performing a full assessment of eTVUS with or without TA, and 32.4% reported performing a partial assessment of eTVUS with or without TA.

Overall, the uptake of eTVUS by Australian sonographers was arguably good. However, full assessment of eTVUS (as per the IDEA consensus) [8, 9] was only performed confidently and routinely by less than a quarter of the participants (22.8%). This study revealed a diverse range of ultrasound assessments being performed for endometriosis detection and called eTVUS, as many respondents reported that they only perform certain aspects of eTVUS rather than a full assessment of eTVUS. This suggested inconsistency in the definition of an eTVUS protocol. The current Australian guideline from the ASUM [7] suggests looking for endometriosis in a gynaecological scan; however, not as a routine inclusion. This could lead to confusion as local departments and individual sonographers are left to guide themselves. Conversely, 30.7% of participants in this study reported that a clear, dedicated local department protocol for the use of eTVUS could facilitate the implementation of this technique into routine practice. Therefore, considering that full assessment of eTVUS was routinely performed by less than a quarter of the participants, the potential benefits of developing a more structured and clearer guideline for Australian departments and sonographers regarding what is expected as an eTVUS assessment for endometriosis diagnosis are highlighted, especially for those outside of OBGYN specialist centres.

When focusing on what was included in a partial eTVUS assessment, the uterine sliding sign (94.6%) and ovarian mobility (97.3%) were the most frequently evaluated. Young et al. [29] reported that evaluations of the sliding sign alone led to a three‐fold increase in the DE detection rate when added to the rTVUS scan. A recent meta‐analysis conducted by Alcázar et al. [30] reported that a negative uterine sliding sign exhibits 88% sensitivity and 94% specificity for POD obliteration, a sign of severe endometriosis, and 81% sensitivity and 95% specificity for DE within the rectum [31]. In cases where surgical management of endometriosis is planned, preoperative knowledge of POD obliteration and bowel DE is vital to ensure triage to appropriately skilled surgeons, adequate patient counselling, and reduce the risk of incomplete or abandoned surgeries [32, 33]. As such, the inclusion of the sliding sign not only has strong diagnostic value but is also a valuable triage tool.

On the other hand, despite its common clinical application and inclusion in the IDEA consensus, there is limited evidence within the literature regarding the diagnostic accuracy of ovarian immobility as a diagnostic marker for endometriosis. A study by Reid et al. [34] revealed ovarian immobility to exhibit a high association with ipsilateral OE, POD obliteration and pelvic DE nodules. Despite this, the finding only revealed positive predictive values (PPVs) of 27% (left) and 14% (right) [34]. Several other studies have revealed ovarian immobility to have low PPVs for endometriosis, suggesting that evaluating ovarian mobility, without searching for endometriosis nodules, could lead to false positive results for endometriosis [12, 35].

A full eTVUS approach includes assessments to search for endometriosis nodules within the anterior and posterior compartments of the pelvis as this allows for direct visualisation of the disease to facilitate diagnosis [8, 9]. Specifically, the USLs and the rectosigmoid colon within the posterior compartment are the areas where DE most commonly presents, and TVUS has been shown in the literature to have good diagnostic accuracy in assessing for DE in these locations [10, 11, 36, 37]. However, in our study, only 37.8% of participants reported assessing the USLs and the rectosigmoid colon for nodules when conducting a partial eTVUS. An inconsistent approach, where the evaluation of these structures is excluded from the assessment, could lead to confusion amongst referrers, reporting clinicians and patients. Managing clinicians may believe their patients are receiving a full endometriosis scan when they are not, which could impact their ongoing clinical management. This may also impact their confidence in utilising eTVUS on their patients.

Within our study, 29.8% of participants reported that their local department allocates insufficient examination time to perform an eTVUS scan. This may be because the examination time required to perform a comprehensive eTVUS in line with the IDEA consensus is, on average, 71% longer than that for an rTVUS [19]. This, along with the additional time that may be required by general sonographers and sonologists to perform and report eTVUS in a community radiology setting, may explain why, in some centres, eTVUS is limited to the assessment of organ mobility only. Recently, Deslandes et al. [38] proposed a simplified eTVUS approach intended for cases where endometriosis is clinically suspected in community settings, which only focuses on the most high‐value anatomical structures of a full eTVUS assessment (uterine sliding sign, USLs, POD and upper rectum). Similarly, early in 2024, a consensus statement was proposed by the Society of Radiologists in Ultrasound (SRU) suggesting an augmented pelvic ultrasound (APU) approach [39]. The SRU proposed augmenting the rTVUS with the addition of ovarian mobility, the uterine sliding sign, and collecting cine clips of the posterior compartment, as well as some optional manoeuvres, for the objective of providing a suggestion of endometriosis which could lead on to specialised comprehensive eTVUS or MRI [39]. Although both approaches were published after the online survey concluded and therefore could not be explored in our study, the findings of our study suggest a simplified approach is probably of value to Australian sonographers, particularly those in general imaging settings. These proposed methods may increase the detection rate of endometriosis while reducing complexity, maintaining a reasonable learning curve and minimising the additional time required compared to a full eTVUS, which would be beneficial to further expand the uptake of eTVUS. It should also be noted that at this stage, both the simplified eTVUS [38] and APU [39] are proposed opinions only, and the diagnostic accuracy of both is currently unknown as neither has been tested in diagnostic accuracy studies.

The results highlighted a lack of departmental mentors for eTVUS (42.3%) as one of the main barriers to eTVUS implementation. Unsurprisingly, participants who were encouraged to perform eTVUS by their local departments, and those who received support from colleagues to provide feedback on their eTVUS technique, were more confident in performing eTVUS than those who did not receive workplace support. Fraser et al. [17] demonstrated that a confident, eTVUS‐trained sonographer could increase the detection rates of endometriosis in the ovaries, retrocervical area and rectosigmoid colon from 25% to 72.5%, 0% to 60% and 5% to 72.5%, respectively, compared to an rTVUS. Therefore, although there is a challenging learning curve associated with eTVUS, as indicated by 38.7% of participants in this study, it is likely heightened within general radiology settings, as our study revealed sonographers in these practices were significantly less likely to report being confident performing eTVUS than those in specialist OBGYN practices. Given the commonality of endometriosis, it is essential that all sonographers have some capability to assess for endometriosis sonographically to help provide patients with valuable imaging. To achieve this, it is necessary for all Australian ultrasound departments that offer gynaecological ultrasound to establish dedicated training programmes and protocols for eTVUS, in addition to education from external ultrasound organisations. This will be essential to ensure patients receive access to more effective and efficient investigations of endometriosis sonographically [40].

This study discovered that participants working in fee‐charging practices are significantly more likely to be able to perform eTVUS compared to those working in bulk‐billing private practices or public hospitals. Considering that the current Australian Medicare system does not provide additional remuneration for performing eTVUS [41], billing limitations may heavily impact the implementation of eTVUS within Australian sonographic practice. This may lead to further equity issues within the population, as our results suggest endometriosis imaging may not be accessible for those who cannot afford to fill the billing gap via either bulk‐billing private centres or public hospitals.

Concerns regarding the reporting system for eTVUS images were also addressed in this study. The survey found that only 51.5% of the time the additional eTVUS findings were always or mostly reported, suggesting a potential lack of support from reporting clinicians, and this may serve as a barrier in providing patients with sonographic diagnoses of endometriosis. Although we do not know why this occurs, with limited literature focusing on this area, it may be that the barriers experienced by sonographers regarding limited training and guidelines are similarly encountered by reporting clinicians, holding back their confidence in reporting eTVUS studies [42].

As Australian sonographic practice is initiated by an imaging referral from the patients' managing clinician, the first step in performing eTVUS and opening the door to a potential sonographic diagnosis of endometriosis is a request for such imaging. Therefore, the inclusion of relevant clinical information on the referral is crucial and cannot be understated. Our results further emphasise this importance, in which a desire to be guided by the clinical question was reported as the greatest facilitator by the participants. In 2020, an international survey conducted by Leonardi et al. [18] revealed that less than half of Australian obstetrician‐gynaecologists would request eTVUS for their patients with symptoms of endometriosis (48.4%). Additionally, only half believed eTVUS could detect DE in the bowel and USLs [18]. Within our study, 25.2% of participants reported that a lack of or limited number of eTVUS referrals prevented them from performing eTVUS in routine practice. Conversely, 28% of participants indicated that eTVUS requests by referring clinicians facilitated their implementation of eTVUS. These findings, along with previous literature, suggest that further education for referring clinicians on the utility of eTVUS in the diagnostic work‐up of people with symptoms of endometriosis may be a potential pathway to address this challenge in the future and open access to a noninvasive diagnosis method for endometriosis to more patients.

A strength of this study was the rigorous design and solid methodology for internet E‐Surveys. Moreover, the response rate was sufficient to achieve a 90% confidence interval, increasing the precision and accuracy of the results. Limitations of this study included the potential sampling bias due to the convenience sample used in this open survey and that the survey could not be sent to all Australian sonographers. According to the most recent Australasian Sonographers Association (ASA) Industry Report, there is approximately one OBGYN‐specialised sonographer for every seven general sonographers in Australia [43]. As such, our survey had a slight overrepresentation of OBGYN sonographers (20.8% of our respondents versus 77.6% general sonographers), suggesting a slight sampling bias. Other limitations include a reduced number of responses on some questions, which limits the generalisability of the study. This study is focused only on sonographer practice, so understanding of some barriers, such as the referring and reporting systems regarding eTVUS, is limited.

## 5 Conclusion

The uptake of some aspects of the eTVUS approach by the IDEA group, such as evaluation of the uterine sliding sign and ovarian mobility, has been good among Australian sonographers. However, assessments of the anterior and posterior compartments for endometriosis nodules are less frequently performed. Training provided to sonographers, limited referrals for eTVUS, and scan time limitations were barriers to implementing eTVUS into routine practice. Moreover, sonographers' desire to answer the clinical question when patients present with symptoms of endometriosis, along with a supportive department, collaborative colleagues, detailed referrals and greater confidence in reporting of sonographer's findings could further facilitate this process. More education surrounding eTVUS for sonographers, reporting clinicians, and referrers could help increase uptake into routine practice. Australian‐based guidelines for eTVUS performance and reporting may also help to mitigate against variation in practice.

## Author Contributions

**Xinyu Yang:** study design, data preparation/collection/analysis/interpretation, participant recruitment, original draft written, manuscript preparation/student researcher. **Alison Deslandes:** conception, study design, data analysis/interpretation, participant recruitment, manuscript revision. **Teresa Cross:** study design, data analysis/interpretation, manuscript revision. **Jessie Childs:** study design, data analysis/interpretation, manuscript revision.

## Conflicts of Interest

The authors declare no conflicts of interest.

## Supporting information


Appendix S1.
