# Supplementary material for: Transvaginal Ultrasound for the Diagnosis of Endometriosis: Current Practices and Barriers in Australian Sonographers
Source: Australas J Ultrasound Med. 2025 May 22;28(2):e70003. doi: 10.1002/ajum.70003 (PMC12097491; doi:10.1002/ajum.70003)

# Appendices

## Appendix 1 – Participants information sheet and survey questions


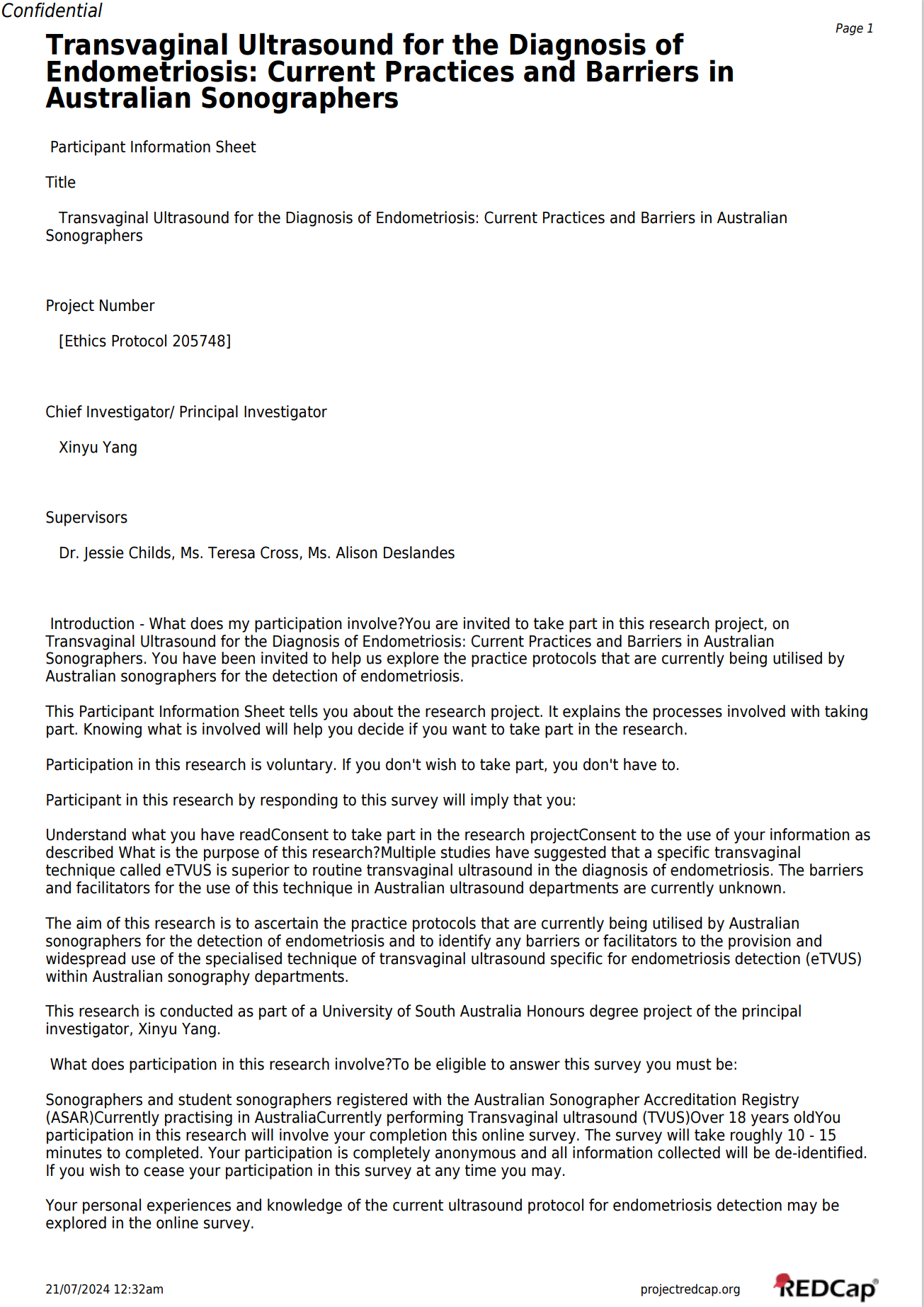


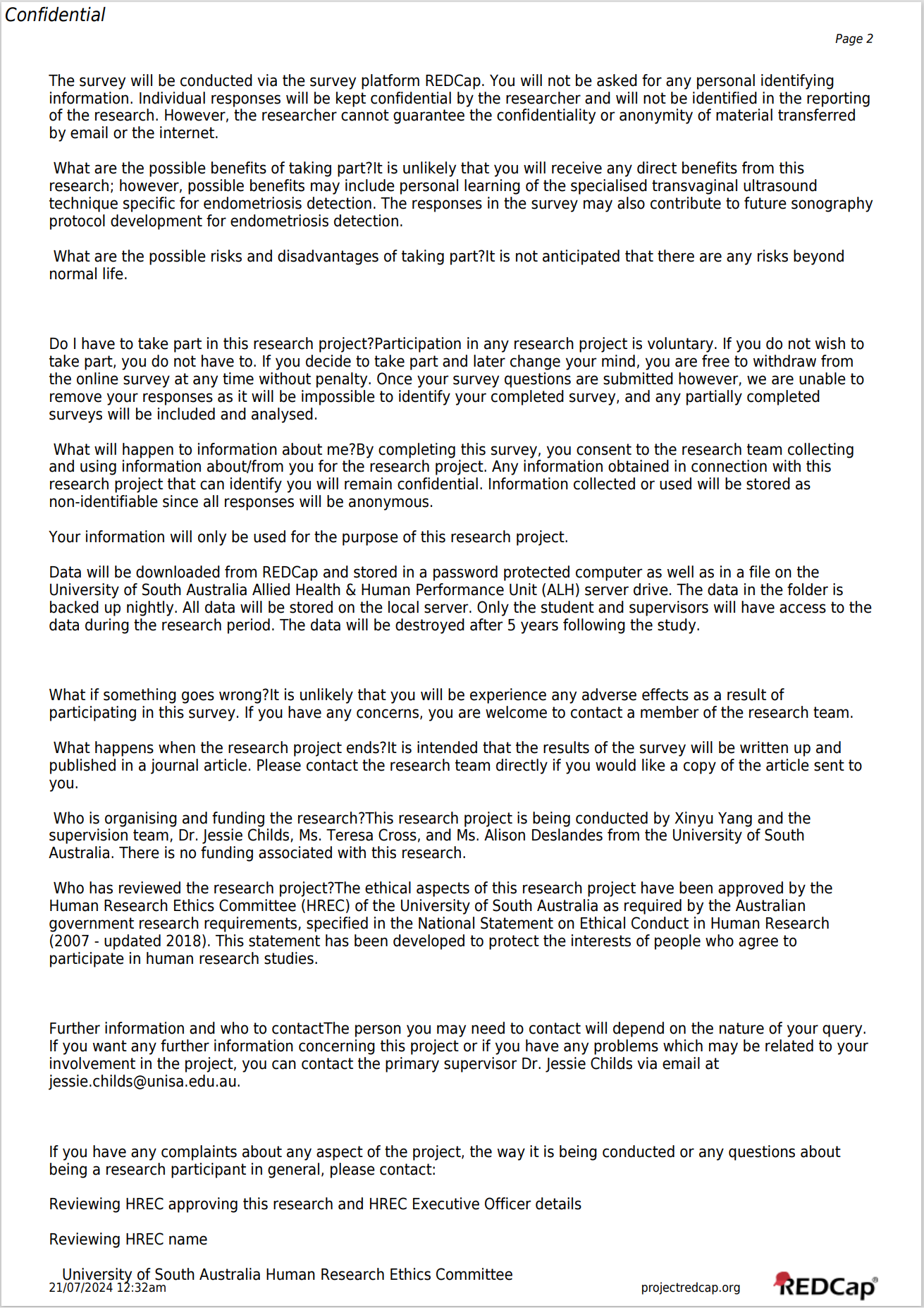


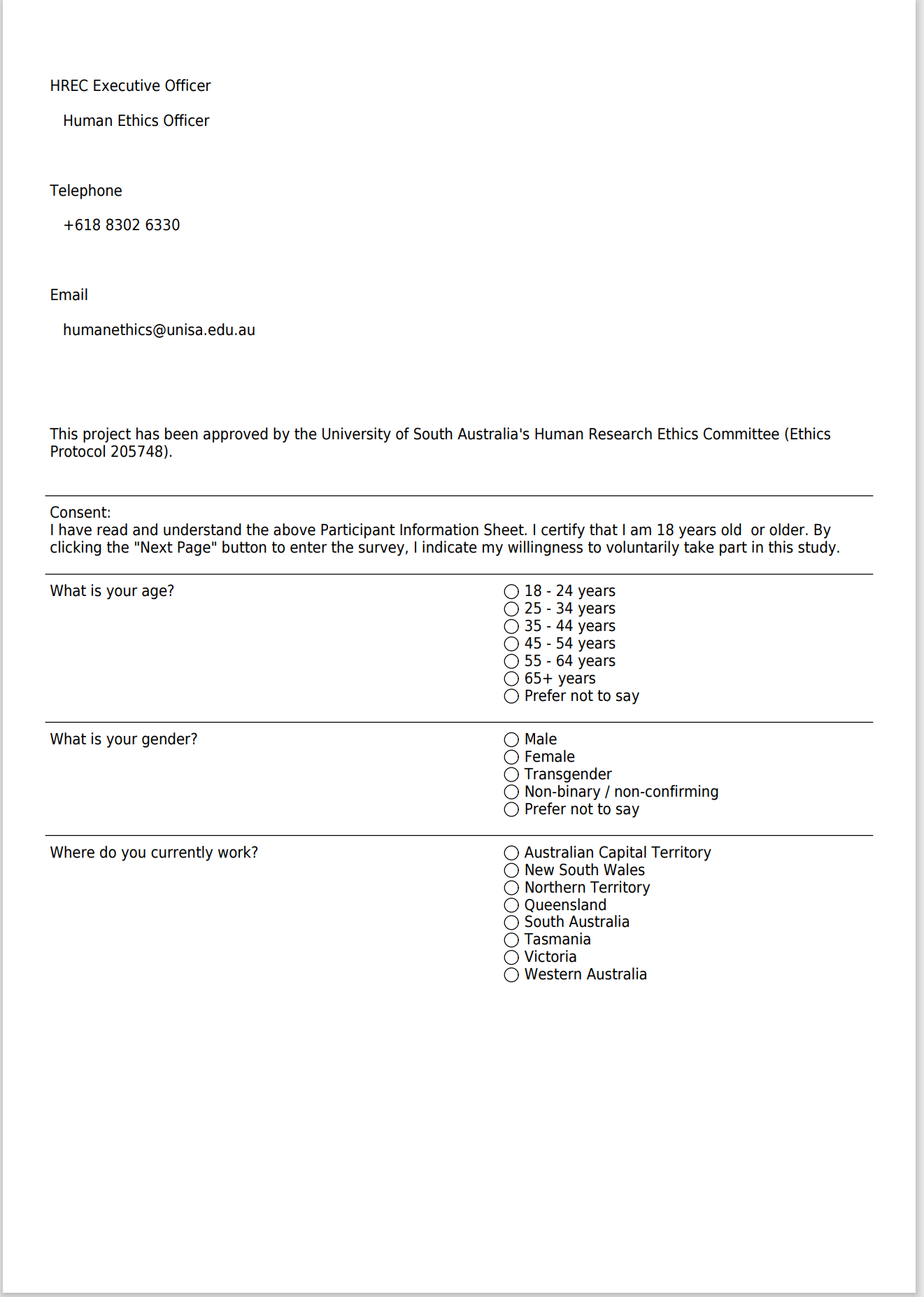


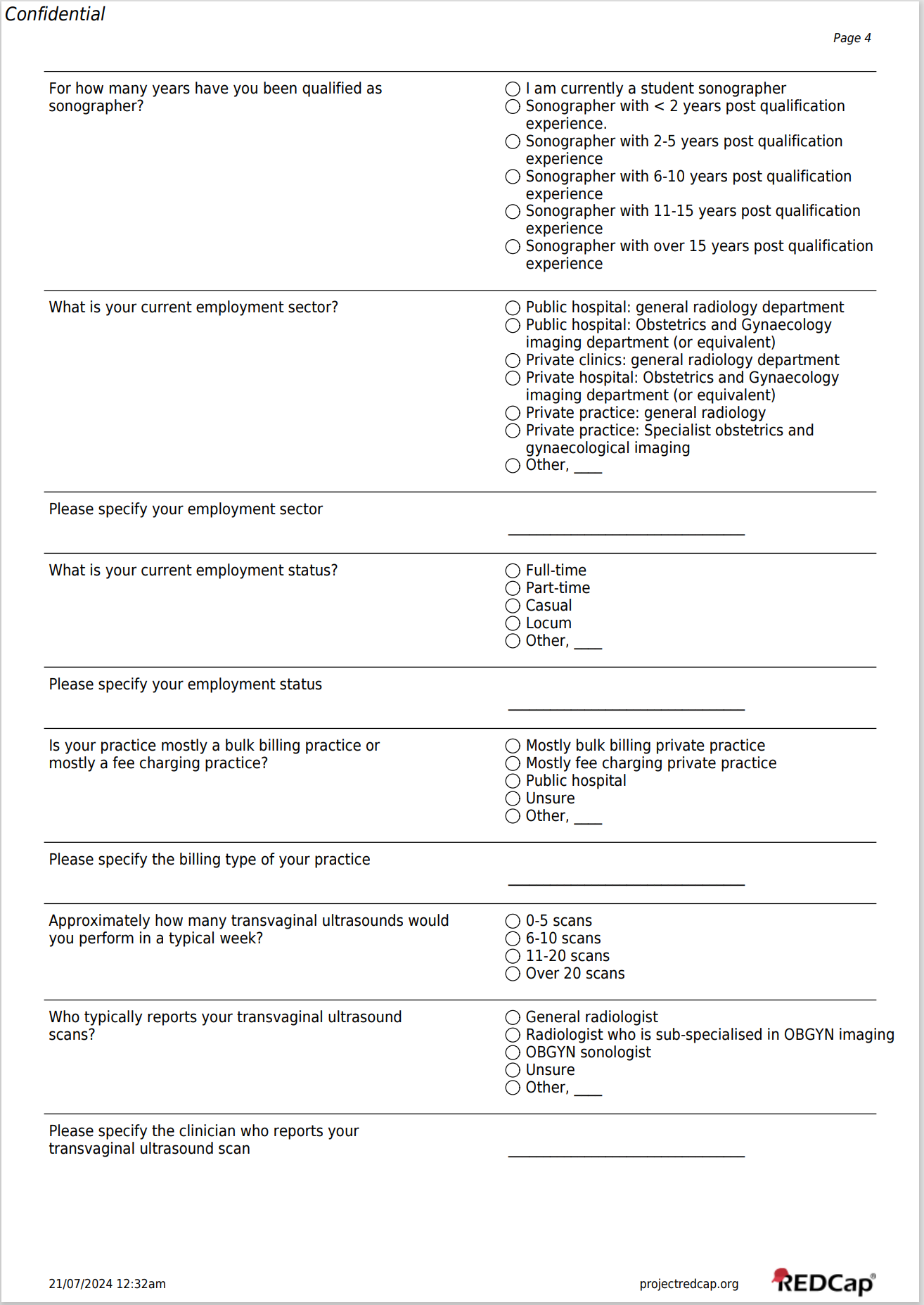


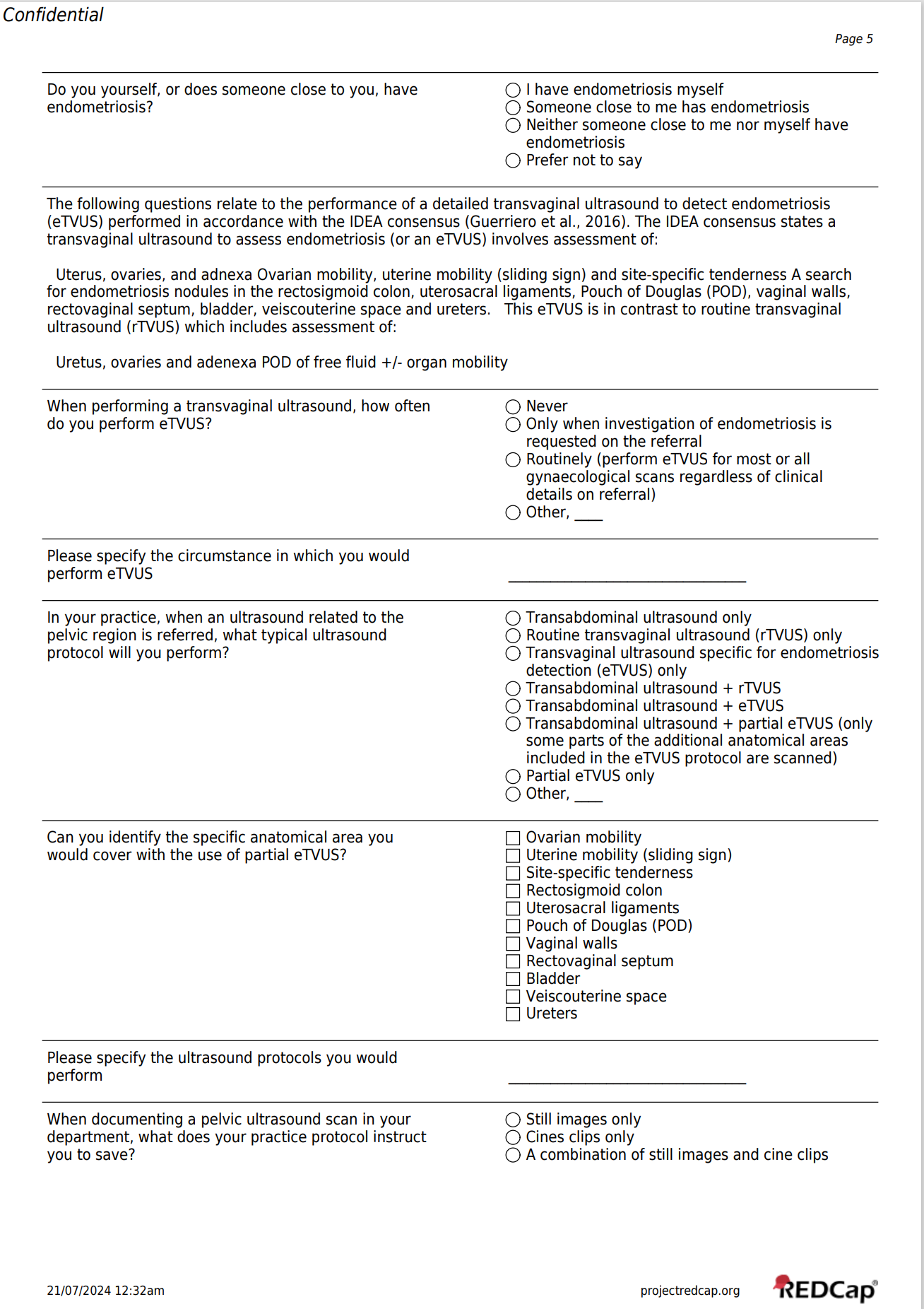


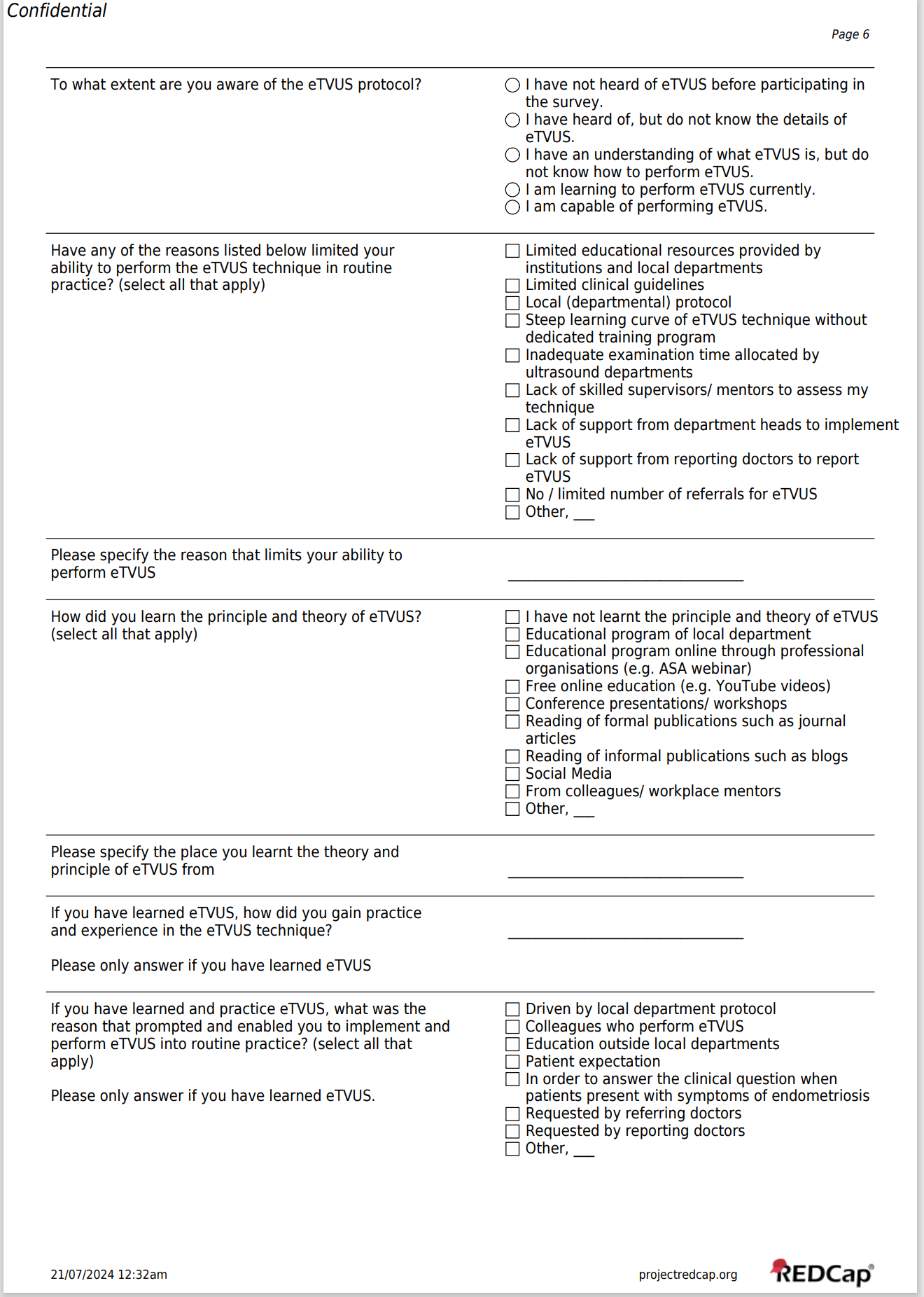


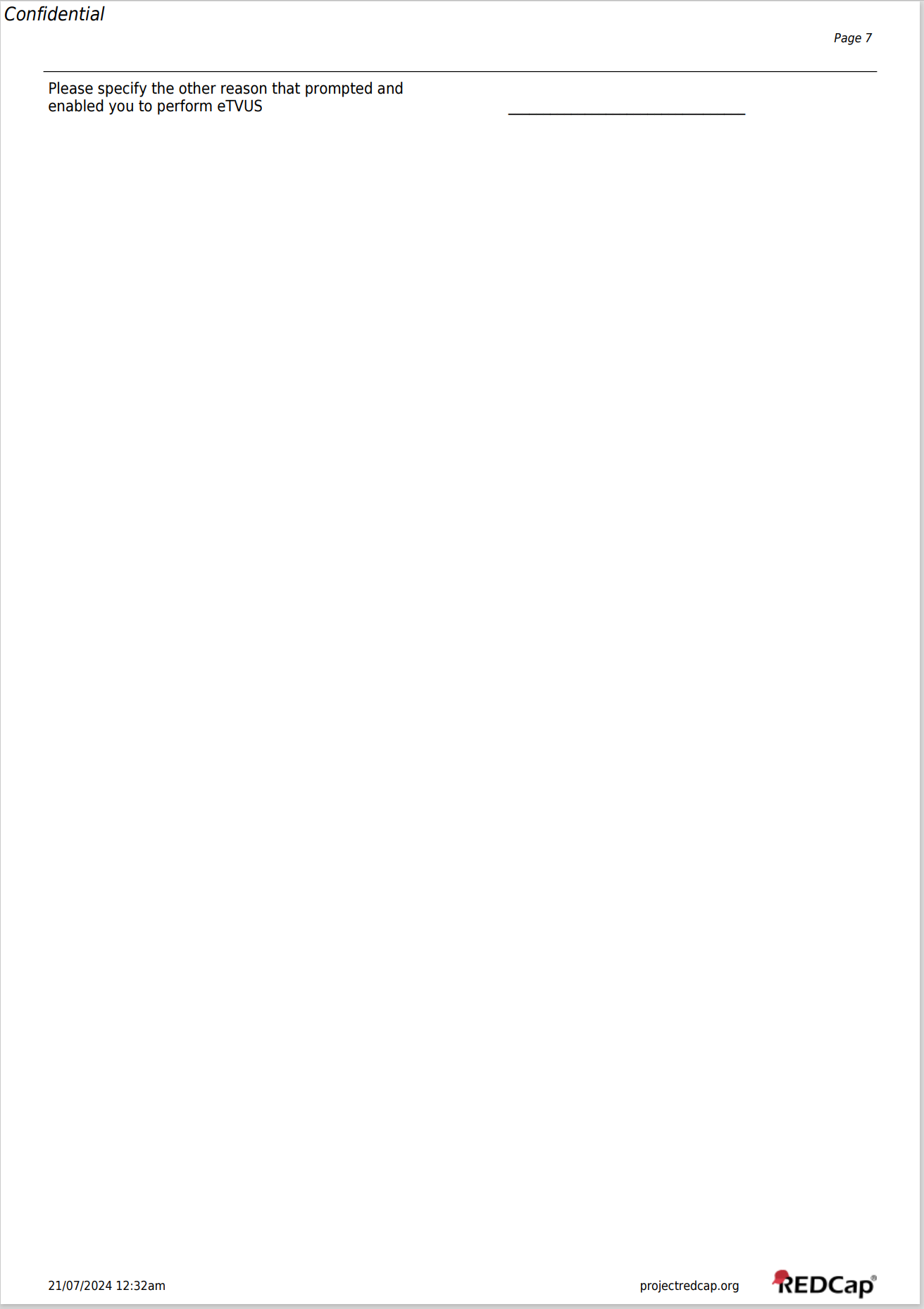


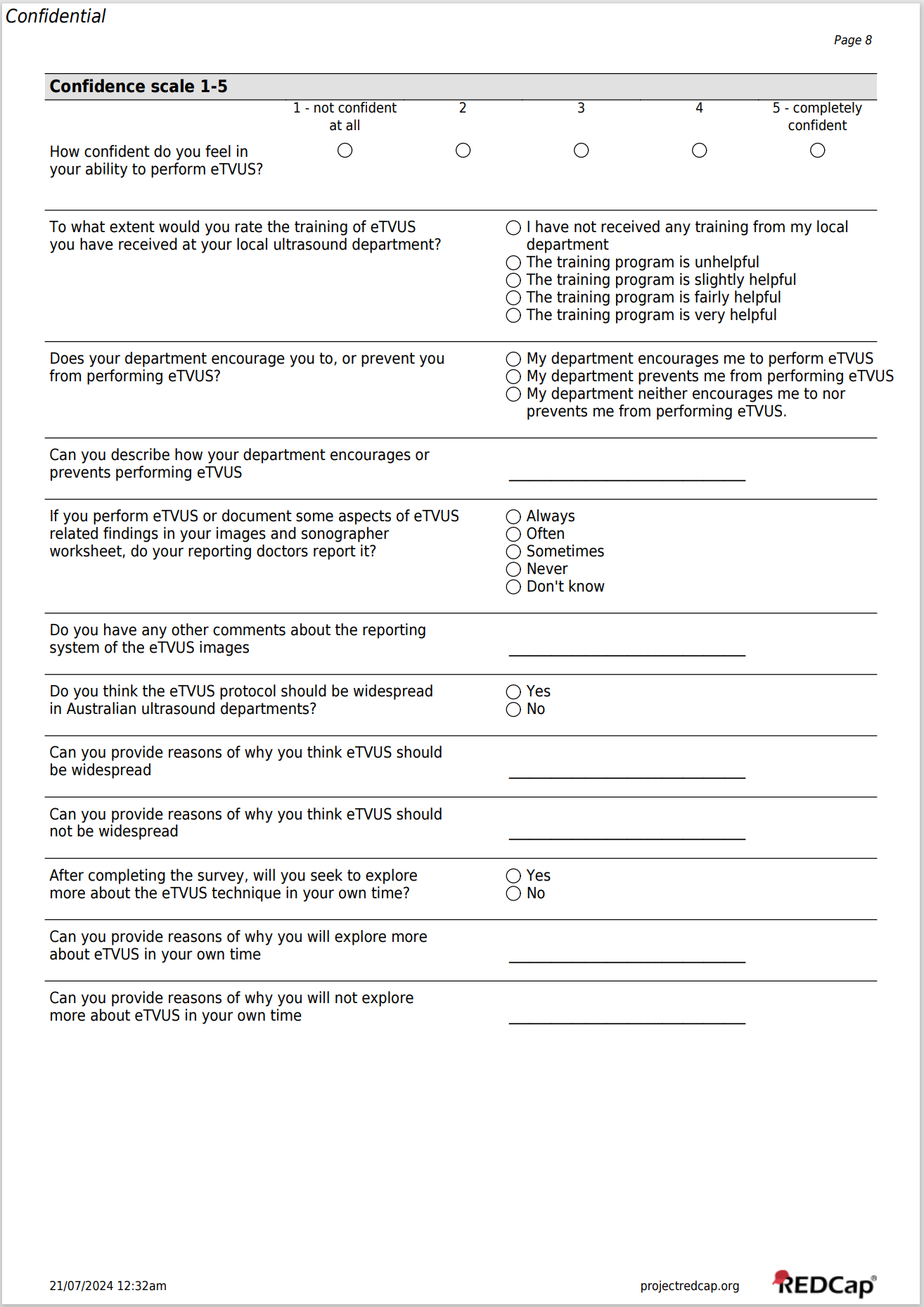


## Appendix 2 – Recruitment material (graphic)


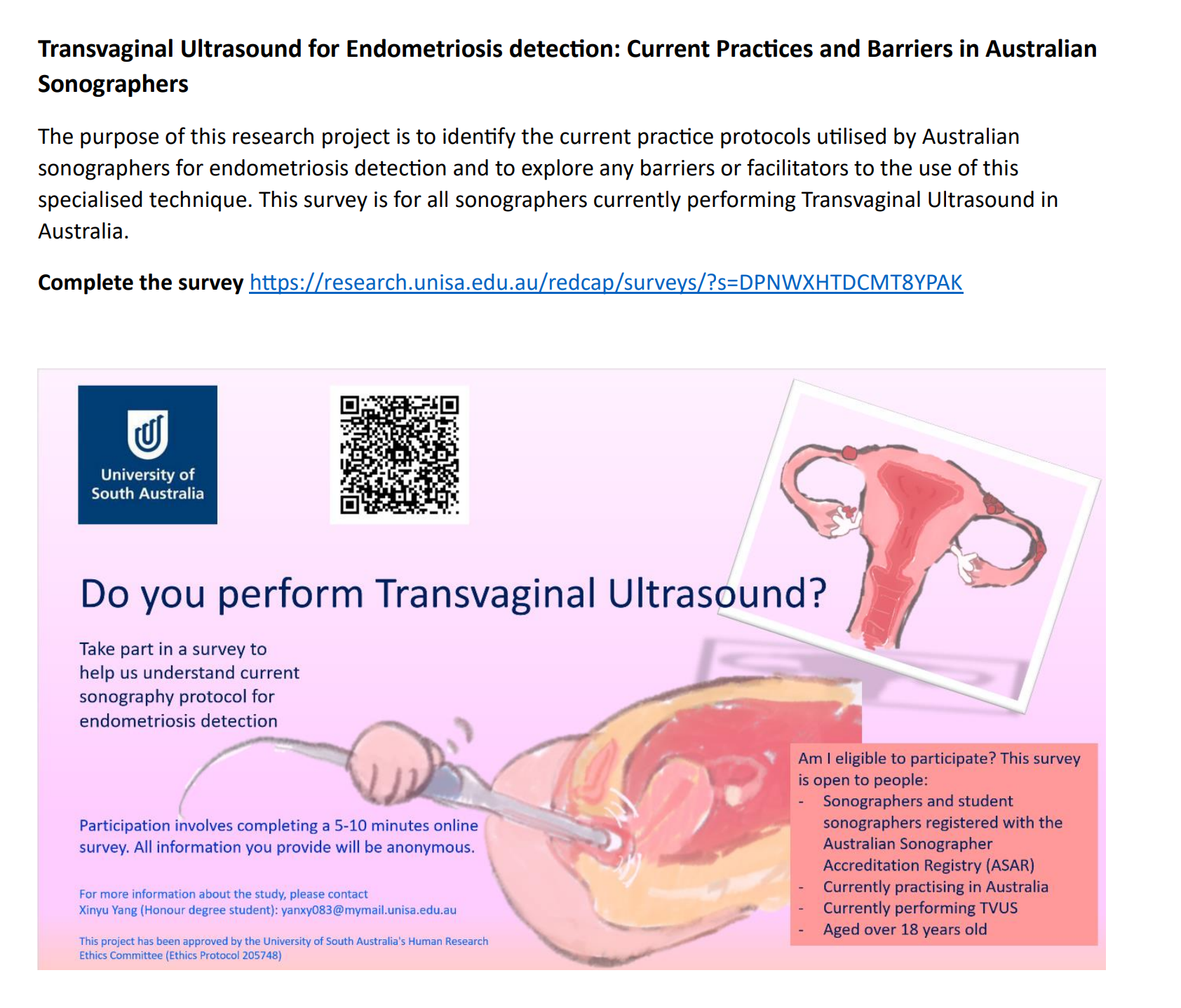

Supplement: Supplementary file 1 — Appendix S1. [file AJUM-28-0-s001.docx]
